# Functional Significance of Aurora Kinases–p53 Protein Family Interactions in Cancer

**DOI:** 10.3389/fonc.2016.00247

**Published:** 2016-11-25

**Authors:** Kaori Sasai, Warapen Treekitkarnmongkol, Kazuharu Kai, Hiroshi Katayama, Subrata Sen

**Affiliations:** ^1^Department of Molecular Oncology, Okayama University Graduate School of Medicine, Dentistry and Pharmaceutical Sciences, Okayama, Japan; ^2^Department of Translational Molecular Pathology, The University of Texas MD Anderson Cancer Center, Houston, TX, USA

**Keywords:** Aurora kinases, p53 tumor suppressor protein family, chromosome instability, centrosome amplification, pluripotency, tumorigenesis

## Abstract

Aurora kinases play critical roles in regulating spindle assembly, chromosome segregation, and cytokinesis to ensure faithful segregation of chromosomes during mitotic cell division cycle. Molecular and cell biological studies have revealed that Aurora kinases, at physiological levels, orchestrate complex sequential cellular processes at distinct subcellular locations through functional interactions with its various substrates. Aberrant expression of Aurora kinases, on the other hand, cause defects in mitotic spindle assembly, checkpoint response activation, and chromosome segregation leading to chromosomal instability. Elevated expression of Aurora kinases correlating with chromosomal instability is frequently detected in human cancers. Recent genomic profiling of about 3000 human cancer tissue specimens to identify various oncogenic signatures in The Cancer Genome Atlas project has reported that recurrent amplification and overexpression of Aurora kinase-A characterize distinct subsets of human tumors across multiple cancer types. Besides the well-characterized canonical pathway interactions of Aurora kinases in regulating assembly of the mitotic apparatus and chromosome segregation, growing evidence also supports the notion that deregulated expression of Aurora kinases in non-canonical pathways drive transformation and genomic instability by antagonizing tumor suppressor and exacerbating oncogenic signaling through direct interactions with critical proteins. Aberrant expression of the Aurora kinases–p53 protein family signaling axes appears to be critical in the abrogation of p53 protein family mediated tumor suppressor pathways frequently deregulated during oncogenic transformation process. Recent findings reveal the existence of feedback regulatory loops in mRNA expression and protein stability of these protein families and their consequences on downstream effectors involved in diverse physiological functions, such as mitotic progression, checkpoint response pathways, as well as self-renewal and pluripotency in embryonic stem cells. While these investigations have focused on the functional consequences of Aurora kinase protein family interactions with wild-type p53 family proteins, those involving Aurora kinases and mutant p53 remain to be elucidated. This article presents a comprehensive review of studies on Aurora kinases–p53 protein family interactions along with a prospective view on the possible functional consequences of Aurora kinase–mutant p53 signaling pathways in tumor cells. Additionally, we also discuss therapeutic implications of these findings in Aurora kinases overexpressing subsets of human tumors.

## Introduction

Gain-of-function alterations in the Aurora kinase protein family member, Aurora kinase-A (AURKA), due to amplification and/or overexpression of the gene- and loss-of-function changes in the TP53 tumor suppressor protein have been associated with multiple cellular phenotypes of similar nature, such as centrosome amplification, override of spindle assembly, and DNA damage checkpoint response, aneuploidy, and cellular transformation. Induction of such shared cellular phenotypes consequent to AURKA overexpression or functional inactivation of TP53 as well as reported localization of the two proteins at the centrosomes indicate that AURKA and TP53 (hereafter referred to as Aurora-A and p53) are involved in overlapping signaling pathways regulating the abovementioned cancer-associated aberrant cellular phenotypes through direct or indirect functional interactions (1–5). Evidence in support of this concept first became available following demonstration that p53 could suppress Aurora-A’s oncogenic potential through physiological interaction in transactivation-independent manner in mammalian cells (6). Similarly, *Xenopus* p53 was shown to inhibit Aurora-A kinase activity, indicating that the inhibitory role of p53 on Aurora-A kinase enzyme activity is conserved among vertebrates (7). Later studies have revealed that p53, besides inhibiting the kinase activity of Aurora-A through direct interaction, also regulates Aurora-A function in transactivation-dependent manner, as discussed below.

In addition to the findings mentioned above, a number of studies have identified Aurora kinases regulating p53 function through phosphorylation-mediated posttranslational modification of either p53 protein directly or a p53 interacting protein at multiple residues with each phosphorylation event having distinct functional consequence. Aurora-A phosphorylates p53 at serine 315, facilitating MDM2-mediated p53 ubiquitination and degradation (8), whereas phosphorylation of serine 215 inhibits p53 DNA-binding and transactivation function (9). These findings demonstrated that Aurora-A phosphorylation of p53 negatively regulates p53 tumor suppressor functions, resulting in abrogation of DNA damage checkpoint and induction of cell death responses in Aurora-A overexpressing cells. As a consequence, Aurora-A overexpressing cancer cells with wild-type p53 acquire cellular phenotypes associated with p53 loss-of-function mutant harboring cancer cells. A more recent finding of a novel Aurora-A phosphorylation residue, serine 106 of p53, was, however, reported to have an opposing effect on p53 stability compared with the destabilization effect of Aurora-A-mediated phosphorylation of p53 at serine 315. Phosphorylation of p53 serine 106 was shown to inhibit the interaction of p53 with MDM2 and prolong the half-life of p53 protein (10). Physiological significance of Aurora-A-mediated p53 phosphorylation at serine 106 *in vivo* and its functional implications in Aurora-A overexpressing tumor cells remain unknown. The possibility of enhanced p53 protein stability in Aurora-A overexpressing tumor cells appears intriguing since steady-state levels of Aurora-A and p53 proteins have been reported to be inversely correlated in most human tumors. Molecular characterization studies have shown that serine 215 phosphorylation is associated with loss of serine 33 phosphorylation of p53, mediated by p38 critical for p53 activation stabilization and induction of apoptosis, indicating that Aurora-A mediates cross-talk between N- and C-terminal posttranslational modifications of p53 (11, 12). In addition, Aurora-A also indirectly compromises p53 function by phosphorylating positive and negative regulators of p53, such as hnRNPK and MDM2 proteins, respectively. The RNA-binding protein, such as hnRNPK, is a p53 transcriptional cofactor that promotes gene expression in response to DNA damage and is also a target of MDM2 (13, 14). While Aurora-A-mediated hnRNPK phosphorylation at serine 379 disrupts its interaction with p53 and impairs DNA damage-induced gene expression, MDM2 phosphorylation at serine 166 enhances its protein stability and in turn destabilizes p53 (15–17). These findings demonstrate that Aurora-A is involved in regulating p53 downstream signaling negatively affecting growth arrest and apoptotic response pathways.

Aurora-B has also been shown to interact with and phosphorylate p53 at multiple residues in DNA-binding domain. Similar to the effect of Aurora-A phosphorylation on p53 activity and stability, Aurora-B phosphorylations of p53 at serine 269 and threonine 284 inhibit p53 transactivation activity, whereas phosphorylations at serine 183, threonine 211, and serine 215 accelerate the degradation of p53 through polyubiquitination-mediated proteasome pathway (18, 19). However, these studies have been performed with phosphor mutants of p53 under conditions of ectopic expression in cells and thus physiological relevance of identical *in vivo* phosphorylations have not been well validated. Further investigations of endogenous protein modifications are required to verify the role of Aurora-B-mediated p53 phosphorylations *in vivo* and to determine how Aurora-A and Aurora-B may be coordinately regulating p53 function through the cell cycle. It is worth noting that exogenously expressed p53 colocalizes with Aurora-B at centromeres during mitosis. This observation may be biologically significant since several spindle assembly checkpoint (SAC) kinases such as MPS1/TTK, BUB1, and BUBR1, localized at kinetochores, have been reported to functionally interact with p53 in activating spindle assembly and postmitotic checkpoint response pathways (20–23). In view of these findings and those demonstrating Aurora kinases regulating functions of p53 family proteins, it is likely that varying levels of Aurora kinases in tumor cells influence the extent of deregulations in checkpoint response pathway activation downstream of p53 family proteins in tumor cells. We discuss the role of Aurora kinases–p53 protein family signaling axis in SAC response pathway later in this review.

Aurora-A involvement in regulating p73 function first became evident from a study in which Aurora-A inhibitor treatment or knockdown of Aurora-A in p53-deficient cells induced p73-mediated expression of apoptosis-related genes and also cell death (16). Further investigation revealed that Aurora-A directly interacts with and phosphorylates p73 at serine 235 in the DNA-binding domain, an equivalent site of serine 215 in p53, resulting in loss of its DNA-binding and transactivation activity. As a result, cells become resistant to DNA damage-induced cell death (24). Importantly, this study uncovered that Aurora-A phosphorylation of p73 leads to the formation of a large molecular complex that includes the chaperon protein Mortalin promoting translocation of the Mortalin–p73 complex into cytoplasm. Similar cytoplasmic distribution of Aurora-A phosphorylated p53 at serine 215 in a complex with Mortalin was observed as well. As a corollary to this finding, cytoplasmic distribution of p73 was found to correlate with Aurora-A expression levels in human primary pancreatic cancer tissues. Moreover, consistent with the earlier findings that p73 deficiency causes relaxation of the SAC reflected in the mislocalization of BUB1 and BUBR1 at kinetochores and reduced BUBR1 kinase activity (25–27), Aurora-A phosphorylation of p73 in a constitutive manner was found to facilitate accelerated mitotic progression and exit accompanied with relaxation of SAC due to premature dissociation of the MAD2–CDC20 complex in proliferating cells *in vitro*. SAC inactivation correlated with significant increase in multinucleated cells. These findings indicate that the mitotic checkpoint functions of p53 family proteins are regulated in a complex manner involving Aurora kinase-mediated posttranslational modifications during mitotic progression. It is currently unknown whether p73 reciprocally controls Aurora-A kinase function and if Aurora-B and Aurora-C also regulate p73 function.

**Figure 1:**
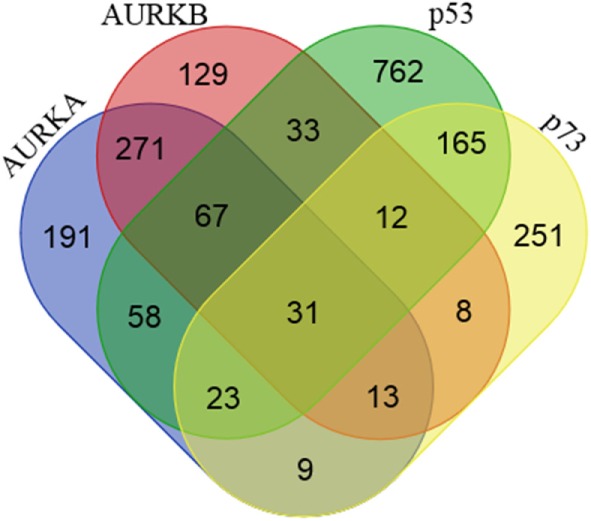
**Proteins interacting with Aurora-A, Aurora-B, p53, and p73**. Venn diagram showing the number of shared and unique proteins interacting with Aurora-A, Aurora-B, p53, and p73. Protein–protein interaction data were downloaded from the BioGRID (v3.4) and STRING (v9.1) databases.

**Figure 2:**
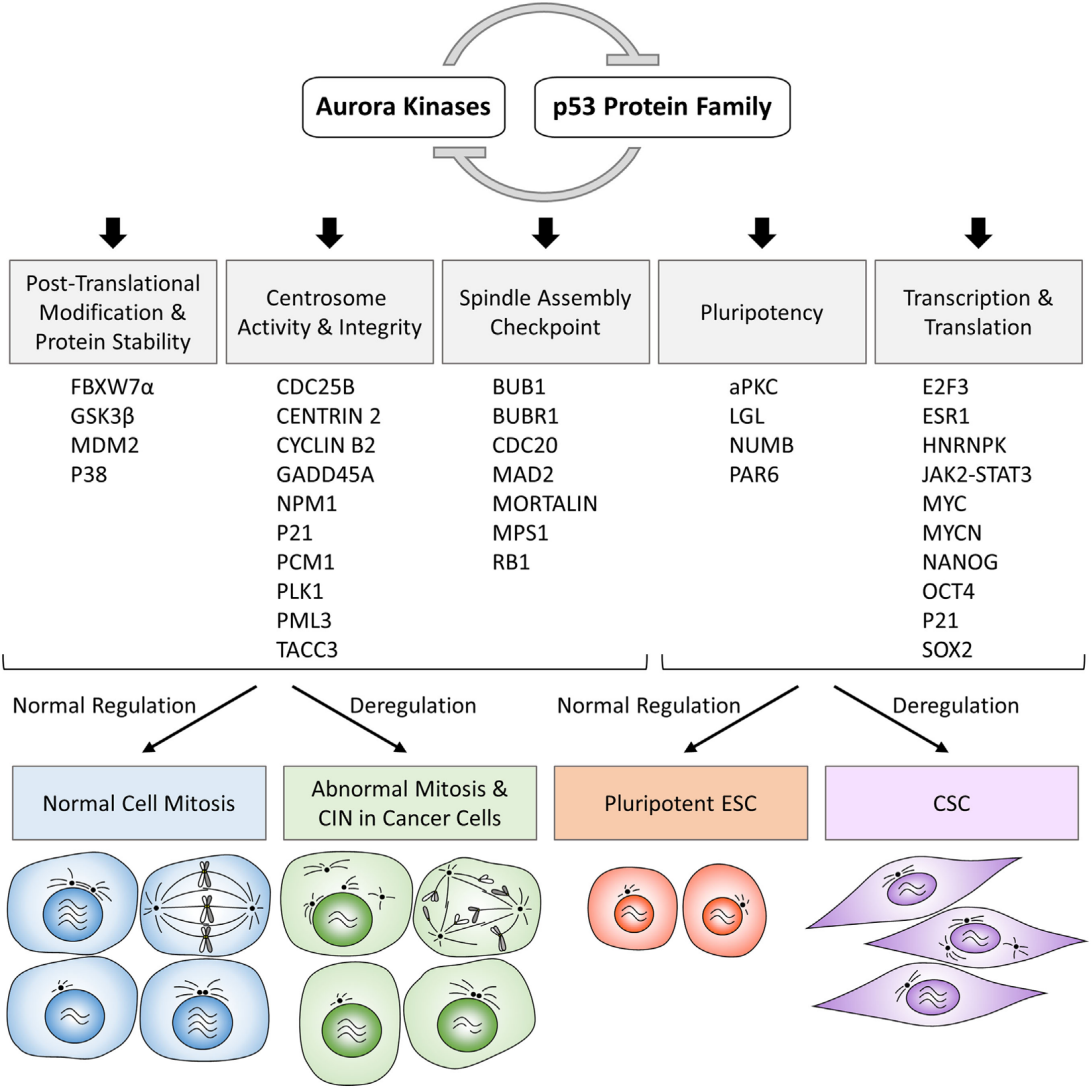
**Schematic overview diagram showing phenotypic consequences of physiologically regulated interactions in normal cells and deregulated interactions in cancer cells involving Aurora kinases–p53 protein family**. CIN, chromosomal instability; ESC, embryonic stem cells; CSC, cancer stem cells.

Along with the discovery of crosstalk between Aurora kinases and p53 family proteins, there is growing evidence that these protein complexes directly or indirectly participate in various cellular processes and inappropriate activation of Aurora kinases can have dominant-negative effects on the phenotypes of normal cells involving pathways regulated by a variety of proteins functionally interacting with p53 protein family (Figure 1; Table 1). In the following sections, we summarize the current knowledge of Aurora kinases–p53 protein family signaling cascades relevant to the regulation of posttranslational modifications and stability of proteins, activity and integrity of centrosomes, checkpoint pathways in normal and aberrant mitosis, as well as protein–protein interactions and transcription and translation of genes involved in the development of pluripotent embryonic stem cells (ESC) and cancer stem cells (CSC), as outlined in the schematic overview diagram in Figure 2.

**Table 1:** **List of proteins interacting with Aurora–p53 family protein complex represented in Venn diagram in Figure 1**.

Combination	Qty	Interacting proteins
AURKA/AURKB/p53/p73	31	ATM, BCL2, BIRC5, BRCA2, CCNA2, CCNB1, CCND1, CCNG1, CDC20, CDC25A, CDC25C, CDK1, CDK2, CDK4, CDKN1A, DDB1, GADD45A, HSPA9, LRPPRC, MTOR, MYL9, PCNA, PTEN, PTTG1, RASSF1, RPS27A, SUMO1, TP63, UBC, UBE2I, XPO1

AURKA/AURKB/p53	67	BARD1, BIRC6, BUB1, CDC14A, CDC14B, CDK5, CENPA, CEP55, DDX5, ECT2, FBXW7, FTH1, FZR1, HNRNPA1, HNRNPU, HSP90AA1, HSP90AB1, HSPA1A, HSPA5, IQGAP1, IRS4, MAP9, NCL, NFKBIA, NINL, NPM1, OFD1, PARP1, PBK, PLK1, PLK3, PPP1CA, PPP1CC, PPP3CA, PRRC2C, PSMB3, PSMC3, PSMC5, PSMD10, PSMD11, PSMD4, PSMD6, PSME3, RPS16, RPS27, RPS4X, RRM2, SETD1A, SMARCB1, TCEAL4, TK1, TOP2A, TOP2B, TP73, TTK, TUBA1A, TUBA1C, TUBA4A, TUBB, TUBB2A, TUBG1, UBA52, UBB, UBE2D1, UBE2N, YY1, YY2

AURKA/AURKB/p73	13	BUB1B, CCNA1, CHFR, E2F2, E2F3, FLT3, HIST1H3C, LATS2, MAD2L1, SASS6, TK2, TP53, TSPO

AURKA/p53/p73	23	AKT1, AURKB, CASP1, CDKN2A, CHUK, CSNK2A1, DICER1, EGFR, ESR1, GSK3B, HDAC2, HRAS, IGF2BP1, IKBKB, MDM2, MYC, NEDD8, PIK3CA, PML, RPL11, RPS19, TAF9, WWOX

AURKB/p53/p73	12	AURKA, BRCA1, CHEK1, CHEK2, DNMT1, EP300, EZH2, H2AFX, HDAC1, MAPK8, PPP1R13L, RB1

AURKA/p53	58	ALB, BTRC, CELA2B, CEP120, CEP128, CEP135, CEP152, CSNK1D, CSNK1E, DCAF7, DGCR14, EEF1A1, EEF2, HAUS1, HNRNPA2B1, HNRNPK, HSPA2, HSPA8, IGF2BP3, ITPKC, KLF4, KRAS, LYZ, MFAP4, MRPL24, MRPS22, NFKB1, NIN, NME1, NRAS, PCMT1, PDCD5, PDCD6, REL, RFC4, RPL12, RPL23, RPL27, RPL30, RPLP0, RPLP2, RPS10, RPS14, RPS3, RPS3A, RPS6, SETD2, SIRT7, SKP1, SRPK1, TFAP2A, TNRC6C, TRIM28, TUBB4B, VHL, YBX1, YWHAE, YWHAG

AURKB/p53	33	ABR, CCDC8, CUL7, DOCK7, DTL, GIGYF2, HDAC5, HDAC9, HERC2, MOGS, MRPS27, MYBBP1A, MYLK, NOC2L, PHKB, PRKDC, RANBP2, RAVER1, RPS25, SKP2, SMARCC1, SNW1, SUMO2, SUMO3, TBC1D4, TUBA8, UBR4, UBR5, UFD1L, VIM, VRK1, WEE1, ZWINT

AURKA/p73	9	AZI1, CCNE1, CDH13, CTNNB1, FUS, MYCN, OAZ1, PRKACA, PSRC1

AURKB/p73	8	ANKRD17, AURKC, CDKN1B, DSN1, GNB2L1, LATS1, STAG1, STK3

## Mechanism of Downregulation of Aurora Kinases by p53

In addition to direct inhibition of Aurora-A by p53 *via* protein–protein interaction, p53 has been shown to downregulate Aurora-A expression, kinase activity and stability through its binding to Aurora-A promoter or transactivation of its target genes including p21, Gadd45a, and Fbxw7α. Genome-wide chromatin occupancy of p53 analyzed by chromatin immunoprecipitation-seq (ChIP-seq) following activation with non-genotoxic molecules and genotoxic chemotherapeutic drugs revealed *AURKA* gene promoter as one of the novel p53 target sequences and that direct p53 binding to the promoter of *AURKA* gene repressed expression in MCF-7 and HCT-116 cells (28). This study also found that STAT3 binds to *AURKA* promoter and antagonizes p53-mediated repression of *AURKA*. Intriguingly, a recent study has shown that Aurora-A promotes STAT3 activity through regulating expression and phosphorylation levels of JAK2 in gastric and esophageal cancers (29), indicating the existence of negative feedback regulation of p53 function by Aurora-A–JAK2–STAT3 axis. These results suggest that the combination of Aurora-A and JAK2 inhibitors with p53 activators might be an effective therapeutic approach for the treatment of cancer. Both p21 and Gadd45a are transcriptionally activated by p53 upon DNA damage and play important roles in DNA repair and cell cycle checkpoint response. The E2F family transcription factor, E2F3 is known to be involved in the transactivation of Aurora-A gene expression during G2–M cell cycle progression (30). Induction of the cyclin-dependent kinase inhibitor, p21 leads to inhibition of Cdk kinase activity resulting in the maintenance of RB1 in hypo-phosphorylated state in a complex with E2F3, thereby impairing activation of Aurora-A gene expression, an indirect downstream effect of p53–p21 signaling axis. It is noteworthy that Aurora-B phosphorylates RB1 at serine 780, a known inhibitory phosphorylation site for Cdk4. Thus, deregulation of Aurora-B might lead to Aurora-A overexpression through direct downregulation of both p53 and RB1 functions. In fact, co-occurrence of increased gene expression of both Aurora-A and Aurora-B is observed in some human tumors. On the other hand, Gadd45a inhibits Aurora-A kinase activity *via* direct interaction to prevent cells from Aurora-A-induced centrosome amplification and aborted cytokinesis (31). These results indicate that cooperative inhibition of Aurora-A activity by p53 and Gadd45a is important for cells to maintain centrosome number and chromosomal/genomic stability.

Besides regulating Aurora kinase function through transcription-dependent and -independent mechanisms, p53 also downregulates Aurora-A activity by modulating its degradation pathway. Fbxw7α is a p53-dependent haploinsufficient tumor suppressor protein and a component of the SCF-like ubiquitin ligase complex that targets both Aurora-A and Aurora-B for proteasome degradation (32–34). Fbxw7α is frequently mutated or downregulated in tumors. Importantly, Fbxw7α cooperates with PTEN to regulate Aurora-A degradation *via* the PI3K/AKT/GSK3β pathway and Fbxw7α also preferentially degrades active Aurora-A (33, 35). It has been demonstrated that Aurora-A-mediated centrosome amplification and subsequent induction of aneuploidy is mediated in part through dysfunction of p53–Fbxw7α axis, commonly detected in human tumors and also in mouse models (33, 36). It is relevant in this context to mention that synthetic lethal screening of protein interacting with N-Myc in N-Myc amplified neuroblastoma has identified that Aurora-A stabilizes N-Myc by directing a K48 to K63/K11 switch in its ubiquitylation by Fbxw7α (37). Although this interaction was reported to be independent of Aurora-A kinase activity, recent finding have demonstrated that inhibitor of Aurora-A kinase activity can disrupt interaction between Aurora-A and Fbxw7α, leading to N-Myc destabilization and tumor regression in mouse model of N-Myc-driven neuroblastoma xenograft (38). Similarly, Aurora-B inhibitor treatment also showed profound growth inhibition and tumor regression in N-Myc-driven neuroblastoma, although the underlying mechanism of this finding remains unclear (39, 40).

Recent studies have identified an important role of microRNA functional networks in the control of gene expression and protein stability of Aurora-A and Myc involving the p53–Fbxw7α axis in neuroblastoma and other tumors. A well-characterized tumor suppressor micoRNA, let-7, regulated by p53 directly targets Aurora-A, c-Myc, N-Myc, and RAN-binding protein 2 (RANBP2). In normal cells, let-7-mediated suppression of c-Myc expression helps maintain basal low level expression of Aurora-A mRNA, while miR-25-targeted Fbxw7α regulates basal level protein expression (41–45). In p53-deficient and p53-mutant cells, these regulatory mechanisms are disrupted, and Aurora-A expression and stability are elevated. Functional genomic studies in N-Myc-amplified neuroblastoma have revealed that LIN28B RNA-binding protein promotes RAN level by directly binding to RAN mRNA and *via* RANBP2 by inhibiting let-7 expression, consequently facilitating Aurora-A activation and stabilization which in turn promote N-Myc stabilization (44). It was recently been reported that Aurora-A acts as a transactivating factor for hnRNPK, a known transcriptional cofactor of p53, to promote c-Myc expression and reciprocal c-Myc-mediated transactivation of Aurora-A gene in breast cancer stem-like cells (46). This finding on apparent absence of p53 inhibitory role in Aurora-A–c-Myc positive regulatory circuit is associated with frequent observation of centrosome amplification in N-Myc-amplified neuroblastoma cells compared to non-amplified neuroblastoma cells. Mechanistically, N-Myc directly transactivates MDM2 and Aurora-A stabilizes MDM2 by phosphorylating at Ser-166 both of which impair p53 function, resulting in centrosome amplification (17, 47, 48). Taken together, these data indicate that p53 controls Aurora-A function through multiple inhibitory signaling pathways and lack of p53 function results in deregulation of Aurora-A oncogenic signaling cascades which lead to profoundly aberrant phenotypes associated with tumor cells. Involvement of additional signaling pathways regulating centrosome activity and integrity mediated by Aurora-A–p53 interaction is discussed below.

## Involvement of Aurora-A–p53 Signaling Pathway in Centrosome Activity and Integrity

**Figure 3:**
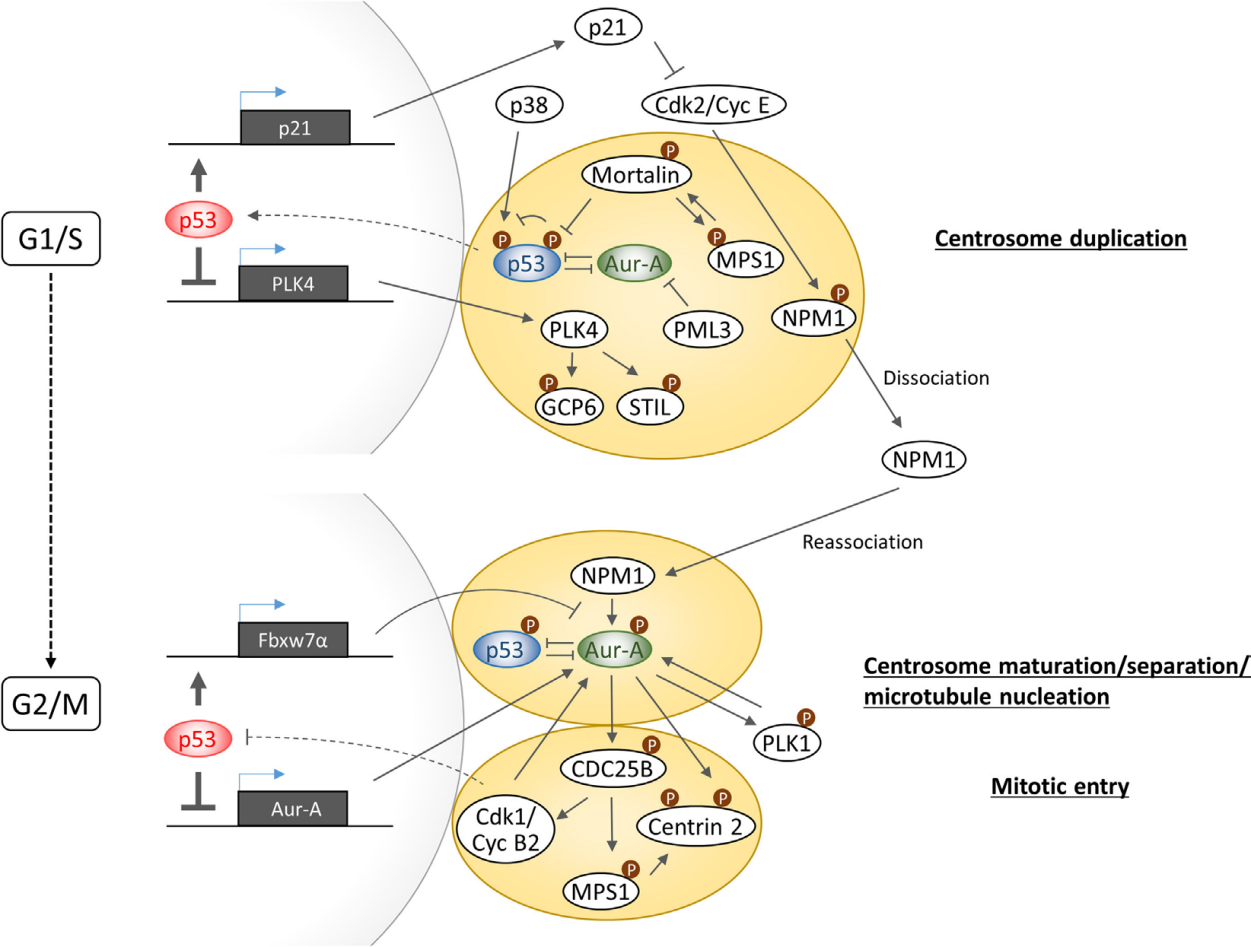
**Schematic diagram illustrating the complexity of Aurora-A–p53-mediated signaling in centrosome biogenesis**.

A common phenotypic change in cells with gain of Aurora-A and loss of p53 function is manifested in the form of increased number of centrosomes. Multiple investigations have revealed that p53 controls centrosome duplication and separation in both transactivation activity-dependent and -independent manner (Figure 3). In transactivation activity-dependent mechanism, p21 expression plays a key role in synchronizing DNA replication and centrosome duplication by inhibiting Cdk2/Cyclin E activity which phosphorylates Nucleophosmin/NPM1 at centrosomes to promote its dissociation from the centrosomes to allow initiation of centrosome duplication (49). On the other hand, p53 downregulates PLK4 gene expression which is essential for centriole biogenesis through regulation of phosphorylations of centrosomal protein GCP6 and STIL (50–52).

In transactivation activity-independent mechanism, centrosomal localization of p53 appears to be critical for negatively regulating centrosome biogenesis and its dissociation from centrosome appears to be sufficient to initiate centrosome duplication. p38–p53 axis was reported to play a central role in inhibition of G1–S cell cycle progression in response to loss of centrosome integrity. Centrosome perturbation caused by depletion of centrosomal proteins such as PCM1, centrobin, and TACC3 promotes the recruitment of both p38 and p53 to centrosomes and facilitate p53 phosphorylation by p38 at serine 33, which in turn transduces the inhibitory signal for cell cycle arrest by inducing p21 expression (53–55). However, the precise function of phosphorylated p53 on centrosome and the molecular mechanism of signal transduction from impaired centrosomes to the nucleus remain unknown. Regarding the mechanism of p53 dissociation from centrosome, a study has revealed that Mortalin through binding to p53 facilitates dissociation of p53 from centrosomes, which in turn results in release of the p53-mediated suppression of centrosome duplication (56). Interestingly, centrosome localization of Mortalin depends on the presence of centrosomal MPS1 kinase which is implicated in the regulation of centrosome duplication and mitotic spindle checkpoint response (57). MPS1 phosphorylates Mortalin, which in turn hyperactivates MPS1 kinase in a feed-forward regulatory manner. Importantly, Mortalin phosphorylation-activated MPS1 can drive centrosome overduplication. Although MPS1 phosphorylation of p53 positively regulates postmitotic checkpoint response (20), the precise role of MPS1 in the regulation of p53 function at the centrosome remains uncertain. Interestingly, the promyelocytic leukemia gene 3 (PML3) was shown to physically interact with Aurora-A and inhibit its kinase activity, while loss of PML3 shown to increase Aurora-A kinase activity and reduced protein stability of p53 along with decreased p21 expression, leading to activation of Cdk2/Cyclin E activity (58). Therefore, since there is no direct evidence supporting a role of centrosome localized Aurora-A in centrosome duplication, it would be imperative to further investigate whether or not increased p53–Mortalin interaction mediated by Aurora-A promotes p53 dissociation from centrosome and accompanying reduction of serine 33 phosphorylation is a cause of centrosome amplification induced in Aurora-A overexpressing cells.

At G1–S transition phase, Nucleophosmin/NPM1 is dissociated from unduplicated centrosome and at G2 phase is again recruited to duplicated centrosome to activate Aurora-A through phosphorylation of serine 89 (59). Activated Aurora-A cooperates with PLK1 to produce the onset signal for entry into mitosis as well as centrosome maturation. Since PLK1 has been shown to induce p53 degradation through phosphorylation of Topors (60), Aurora-A–PLK1 functional interaction, therefore, could interfere with p53 function on the centrosome at G2/M phase. NPM1-activated Aurora-A has also shown to induce phosphorylation of Centrin 2 at serine170 for stabilization of the protein (61). Phosphorylation of CDC25B at serine 353, which in turn stabilizes MPS1, also leads to stabilization of Centrin 2 through phosphorylation (62, 63). These findings indicate that Aurora-A and MPS1 cooperatively regulates Centrin 2 stability to induce centrosome maturation and separation. Activation of CDC25B is also pivotal for activation of Cdk1/Cyclin B, and a recent study has revealed that Cyclin B2 antagonizes p53 inhibitory activity against Aurora-A to control proper timing of centrosome separation at the onset of mitosis (64). Taken together, Aurora-A signaling branches off from CDC25B toward MPS1 for control of Centrin 2 stabilization regulating centrosome activity and toward Cdk1/CyclinB for positive feedback toward activation of Aurora-A in part by preventing p53 inhibitory action on Aurora-A.

The studies mentioned above clearly present evidence in support of a critical role for p53 signaling in regulating centrosome biogenesis and activity through cell cycle. In view of Aurora-A expression levels correlating with centrosome number and activity as well as known Aurora-A functional interactions with p53, Mortalin, PLK1, CDC25B, and, possibly MPS1, it will be interesting to investigate how the entire signaling axis involving these proteins maintains centrosomal homeostasis in proliferating cells.

## Aurora-A–p73 Interaction in Spindle Assembly Checkpoint

**Figure 4:**
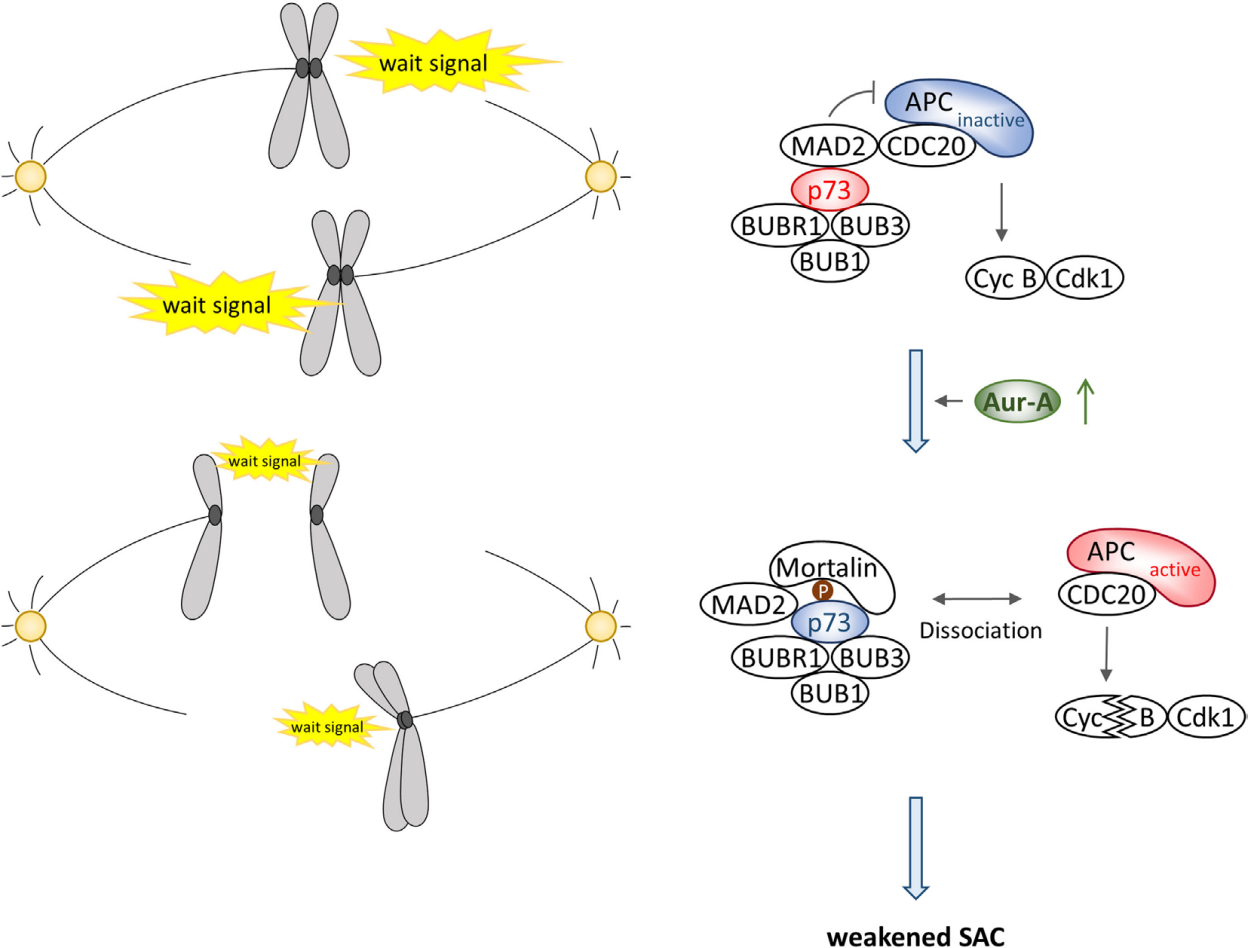
**Schematic illustration of Aurora-A–p73 interaction in spindle assembly checkpoint**.

A number of studies have shown the association of deregulated Aurora-A expression and activity with SAC override in cells irrespective of the p53 functional status in cells. Therefore, it is currently unclear whether or not p53 is involved in Aurora-A-mediated signal for SAC override. Accumulating evidence consistently suggest that p53 also functions in mitotic cell death and postmitotic checkpoint activated following aberrant mitosis and/or spindle damage through interaction with and phosphorylation by SAC proteins rather than being involved in the activation of SAC (20, 65–68). On the contrary, the role of Aurora-A–p73 interaction in SAC is relatively better defined. *In vitro* studies have shown roles of p73 in G2–M transition, mitotic exit, and mitotic cell death (69–72), while analysis of transgenic mouse lacking transactivation competent p73 (TAp73) revealed frequent occurrence of aberrant spindle structure associated with aneuploidy, chromosome instability, and mitotic slippage with spindle poisons (26). Further biochemical studies have also shown interaction of TAp73 with SAC proteins BUB1, BUB3, and BUBR1, and this interaction is crucial for BUB1 and BUBR1 localization at kinetochores and BUBR1 kinase activity (26, 27). These results suggest that TAp73 is directly involved in regulating SAC pathway to maintain chromosome stability. More recent study has demonstrated that TAp73 interacts with the inhibitory mitotic checkpoint complex of MAD2 and CDC20, preventing activation of the E3 ubiquitin ligase APC/C, and that Aurora-A phosphorylation of TAp73 at serine 235 causes dissociation of the MAD2–CDC20 complex, facilitating mitotic exit (24), suggesting that Aurora-A–TAp73 interaction is essential for a critical step in the SAC inactivation pathway (Figure 4). Unlike its effect on MAD2–CDC20 interaction and p73 depletion induced mislocalization of BUBR1 from the kinetochore, phosphorylation of p73 does not affect interaction of BUBR1 with CDC20 and its kinetochore localization, indicating that p73 participates in distinct pathway to control SAC activation. Although serine 235 phosphorylation of p73 enhances its interaction with Mortalin as described above, a more detailed investigation on the role of Aurora-A–Mortalin signaling axis in mitotic progression and SAC is warranted.

Expression level of transactivation-defective ΔNp73 is known to be elevated in many tumors and ectopic expression of transactivation-defective ΔNp73 has been implicated in abnormal mitotic progression accompanied with multipolar spindle and cytokinesis failure resulting in multinucleated cells. However, ΔNp73 neither affects SAC activation in the presence of spindle poison nor is it known to interact with BUBR1 (26, 73), indicating that expression of ΔNp73 helps bypass SAC. Intriguingly, Aurora-A also interacts with and phosphorylates ΔNp73 with similar efficacy as that of TAp73 but its phosphorylation site is different from TAp73 that remains to be mapped (24). Thus, characterization of physiological role of Aurora-A phosphorylated ΔNp73 could provide evidence of a novel signaling pathway affecting SAC.

## Aurora-A–p53 Signaling in Pluripotent Cells

Aurora-A has been reported to suppresses p53 function *via* phosphorylation of cell-fate determinant protein NUMB. While NUMB interacts with and helps stabilize and activate the tumor suppressor protein p53 (74, 75), Aurora-A initiates a phosphorylation cascade of aPKC–PAR6–Lgl cell polarity complex that ultimately leads to NUMB phosphorylation during mitosis to commit to asymmetric cell division (76–78). A recent study has revealed that phosphorylation of NUMB by Aurora–aPKC cascade disrupts its binding to p53 and promotes MDM2-mediated p53 degradation in cancer initiating cells of liver cancer (79). Thus, Aurora-A also antagonizes p53 activity indirectly through aPKC activation, resulting in maintenance of pluripotent state of cells and possibly promoting tumorigenesis. It would be interesting to examine if Aurora-A phosphorylation of p53 and NUMB synergistically affect disruption of their bindings.

A number of studies on cancer stem-like cells have revealed strong association of Aurora-A expression with gene expression of core stemness markers, such as Myc, Sox2, and Oct4. Additionally, Aurora-A–p53 functional interaction in the regulation of self-renewal and differentiation of mouse embryonic stem cells (mESC) and somatic cell reprograming has also been investigated (80, 81). Loss-of-function screening for protein kinases and phosphatases essential in mESC development and subsequent functional studies revealed strong correlation between elevated expression of Aurora-A and the undifferentiated state of mESC. Furthermore, loss of Aurora-A, but not loss of Aurora-A, mitotic substrates compromised self-renewal and triggered differentiation of mESC, indicating that non-canonical function of Aurora-A, unrelated to its role in mitosis, is possibly involved in regulating self-renewal potential of mESC (82). This observation also showed inverse correlation with p53 activity in mESC and attributed this finding to Aurora-A-mediated inactivation of p53 function. The study also revealed that Aurora-A-mediated serine 215 phosphorylation rather than serine 315 phosphorylation is more critical in antagonizing p53-induced mESC differentiation and p53-mediated suppression of induced Pluripotent Stem Cells (iPSC) reprograming *via* activation of gene expression program associated with pluripotency. Phosphorylation of serine 315, on the other hand, was shown to cause partial impairments of both mESC differentiation and suppression of iPSC reprograming correlating with lower expression of pluripotency markers. The varying degree of downstream effects of the two Aurora-A-mediated p53 phosphorylated residues possibly represents stronger inhibition of p53 function following serine 215 phosphorylation resulting in complete loss of its transactivation activity and cytoplasmic sequestration reflecting the naturally observed localization of endogenous p53 in mESC (83). The study concluded that Aurora-A controls pluripotency through inhibition of p53 target gene expression required for ectodermal and mesodermal differentiation. The observation regarding serine 315 phosphorylation showing less pronounced phenotype in this study appeared conflicting to an earlier report showing elevated serine 315 phosphorylation during mESC differentiation and knockin of serine 315 phosphor-deficient mutant impairing mESC differentiation. Importantly, serine 315 phosphorylation was also reported to enable the recruitment of the corepressor mSin3a to the NANOG promotor, resulting in complete suppression of NANOG transcription and primitive endodermal differentiation (84–86). Serine 315 phosphorylation is known to be mediated not only by Aurora-A but also by Cdk/cyclin complex. In view of the observed loss of serine 33 phosphorylation in serine 215 phosphorylated p53, it is plausible that serine 215 phosphorylation might inhibit serine 315 phosphorylation by Cdk1 or Aurora-A. Alternatively, Aurora-A phosphorylation of the two p53 residues may be playing non-overlapping physiological roles in Aurora-A-mediated cellular processes.

In contrast to the requirement of Aurora-A in maintenance of pluripotency and induction of iPSC state mentioned above, a study reported that loss of Aurora-A function is essential for somatic cell reprograming (87). In this study, authors reported that loss of Aurora-A function following small-molecule inhibitor treatment or siRNA knockdown enhanced efficacy of iPSC generation with cells reaching a fully reprogramed state. The iPSC generated by this approach possessed ability to differentiate into different lineages *in vitro* and *in vivo*. Moreover, p53 depletion could further enhance the effect of loss of Aurora-A function. The underlying reasons for these contradictory findings are not known at this time and need to be investigated.

## Genetically Engineered Aurora-A–p53 Targeted Mouse Models

Comprehensive genomic analyses have identified Aurora-A as a low penetrance tumor-susceptibility gene and elevated expression was reported to play an essential pathological in tumor development correlating with prognosis and resistance to therapy (80, 88–91). Several transgenic mouse models have been developed to gain direct evidence of Aurora-A tumorigenic potential and associated phenotypic alternations *in vivo*, which have yielded somewhat conflicting and distinct results (92–94). While Wap-Cre mouse model system in which Aurora-A was constitutively overexpressed under *CAG-CAT* promoter in mammary gland after one cycle of pregnancy developed hyperplasia in p53 wild-type background and precancerous atypical ductal hyperplasia in p53–null background (92, 94), *MMTV* promoter-driven mouse model was reported to develop mammary tumors in both p53 wild-type and heterozygous background after four to five cycles of pregnancy (93). Notably, centrosome amplification and chromosome instability were detected in all mouse models, suggesting that Aurora-A overexpression affects p53 function in the maintenance of centrosome homeostasis and chromosomal stability *in vivo*. Consistent with *in vitro* studies, activation of AKT signaling pathway leading to Cyclin D overexpression was seen in the tumors developed in *MMTV*–Aurora-A mice. We have recently reported a mammary gland targeted Aurora-A mouse model in a p53 wild-type background in which Aurora-A expression is driven by ovine β-*lactoglobulin* promoter led to the development of mammary tumors after four to five of pregnancy cycles (95). In addition to genomic instability, reduced expression of p53 protein and activation of AKT signal pathway was detected in tumors similar to *MMTV*–Aurora-A mouse model, again suggesting that elevated levels of Aurora-A can be oncogenic with inhibitory effects on p53-mediated tumor suppressor signaling pathways. It is relevant to mention, in this context, that an inducible gene switch mouse model overexpressing Aurora-A in skin epidermis exposed to tumor promoter 12-*O*-tetradecanoylphorbol 13-acetate (TPA) and the mutagen 7,12-dimethylbenz(*a*)anthracene, developed by us earlier, revealed malignant progression of skin tumors with centrosome amplification, abnormal spindle formation, and genomic instability (96). Expression of p53 protein was lost, and amplification of MDM2 gene was concurrently found in these tumors. Taken together, Aurora-A overexpressing mouse models of organ-specific tumors have revealed loss of p53 expression recapitulating naturally occurring Aurora-A and p53 expression changes seen in human tumors. Further in-depth studies to elucidate the role of Aurora-A–p53 signaling cascades relevant to human tumor development utilizing Aurora-A overexpressing mouse models are warranted.

## Conclusion

Functional interactions between Aurora kinases and p53 family proteins coordinately regulate diverse cellular pathways by modulating activity and subcellular localization of each other and their downstream effector proteins. Deregulations of these interactions in cells undergoing tumorigenic transformation have significant functional consequences on induction of chromosome instability, development of different tumor-associated phenotypes including resistance to therapy. In addition to Aurora kinase functional interactions with wild-type p53 and p73, there is evidence of Aurora-A interacting with and phosphorylating mutant p53 protein. Physiological function of Aurora-A–mutant p53 interactions have not been elucidated yet. Mutant p53 and transactivation-deficient mutant of p73 also phenocopy some of the Aurora-A overexpression-induced phenotypes. It would be interesting to investigate the functional consequences of Aurora-A phosphorylation of mutant p53 family members in the p53 signaling cascades and their significance in the development of tumorigenic phenotypes.

## Author Contributions

All authors contributed to writing the review and preparing figures.

## Conflict of Interest Statement

The authors declare that the research was conducted in the absence of any commercial or financial relationships that could be construed as a potential conflict of interest.
